# PolyG Fibrils Coalesce Into Nuclear Ribbons That Engage Proteostasis Machinery in Neuronal Intranuclear Inclusion Disease

**DOI:** 10.1002/advs.76630

**Published:** 2026-07-20

**Authors:** Hui Dong, Yongcheng Pan, Zhiyao Tang, Yuxuan Yao, Guicong Zhang, Junpu Wang, Haonan Xiao, Yun Tian, Beisha Tang, Dan Li, Qiang Guo, Ruijun Tian, Qiong Liu, Cong Liu

**Affiliations:** ^1^ Interdisciplinary Research Center on Biology and Chemistry State Key Laboratory of Chemical Biology Shanghai Institute of Organic Chemistry Chinese Academy of Sciences Shanghai China; ^2^ Key Laboratory of Hunan Province in Neurodegenerative Disorders Department of Neurology & National Clinical Research Center for Geriatric Disorders Xiangya Hospital Central South University Changsha Hunan China; ^3^ State Key Laboratory of Medical Proteomics and Shenzhen Key Laboratory of Functional Proteomics Department of Chemistry and Research Center for Chemical Biology and Omics Analysis School of Science and Guangming Advanced Research Institute Southern University of Science and Technology Shenzhen China; ^4^ Bio‐X Institutes Key Laboratory for the Genetics of Developmental and Neuropsychiatric Disorders (Ministry of Education) Shanghai Jiao Tong University Shanghai China; ^5^ Zhangjiang Institute for Advanced Study Shanghai Jiao Tong University Shanghai China; ^6^ Department of Pathology Xiangya Hospital Central South University Changsha Hunan China; ^7^ Department of Geriatrics Xiangya Hospital Central South University Changsha Hunan China; ^8^ State Key Laboratory of Membrane Biology Peking‐Tsinghua Center for Life Sciences Academy for Advanced Interdisciplinary Studies School of Life Sciences Peking University Beijing China; ^9^ Changping Laboratory Beijing China; ^10^ Shanghai Academy of Natural Sciences (SANS) Shanghai Institute of Organic Chemistry Chinese Academy of Sciences Shanghai China

**Keywords:** cryo-electron tomography, Neuronal Intranuclear Inclusion Disease (NIID)

## Abstract

Neuronal intranuclear inclusion disease (NIID) arises from GGC repeat expansions in *NOTCH2NLC*. These expanded repeats produce polyglycine (polyG) proteins, and the accumulation of these polyG proteins in neuronal nuclei serves as the characteristic pathological hallmark of NIID. However, the native cellular ultrastructure of polyG and its contribution to pathology remain poorly understood. Here, using a transgenic NIID mouse model, we extract polyG assemblies from diseased brain and characterize their architecture by cryo‐electron tomography (cryo‐ET). We further examine their native organization by tracer‐guided in situ cryo‐ET in vitrified mouse brain. We find that polyG forms highly branched ∼5 nm fibrils that laterally coalesce into densely packed ribbons, which represent the predominant polyG state within neuronal nuclei in situ. In parallel, proximity‐dependent labeling coupled to mass spectrometry reveals selective enrichment of proteostasis factors—including proteasome subunits and molecular chaperones—at polyG assemblies in mouse brain. Consistent with this, cryo‐ET visualizes proteasome‐like particles decorating ribbon‐shaped surfaces and edges in cells. Together, these findings uncover an unexpected ribbon‐shaped supramolecular architecture for a low‐complexity disease protein and suggest that nuclear polyG ribbons act as scaffolds that engage proteostasis machinery, providing mechanistic insight into NIID.

## Graphical abstract

In NIID, expanded NOTCH2NLC repeats give rise to nuclear polyG inclusions. Tracer‐guided in situ cryo‐electron tomography enables cross‐scale structural analysis from mouse brain to native neuronal nuclei, revealing dense‐core/peripheral‐halo inclusions built from compact polyG ribbons. Proteomics and tomography further show proteasome enrichment on polyG ribbons, linking ribbon architecture to altered nuclear proteostasis.

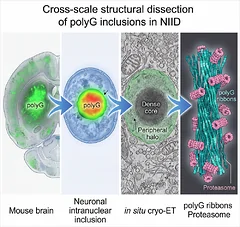

## 1 Introduction

Neuronal intranuclear inclusion disease (NIID) is a progressive neurodegenerative disorder characterized by eosinophilic, ubiquitin‐ and p62‐positive intranuclear inclusions in neurons, glial cells, and multiple peripheral tissues [1, 2, 3]. For decades, NIID is primarily diagnosed through pathological examination of biopsies or autopsies, which reveals its characteristic intranuclear eosinophilic inclusions [4, 5, 6, 7]. In 2019, GGC repeat expansions in the 5′untranslated region (5'UTR) of *NOTCH2NLC* were identified as the causative mutation in both familial and sporadic NIID by several independent research groups [8, 9, 10, 11]. Subsequently, this mutation was identified in several other diseases, including essential tremor, Parkinson's disease, Alzheimer's disease, frontotemporal dementia, multiple system atrophy, adult leukoencephalopathy, amyotrophic lateral sclerosis, cerebral small vascular disease and oculopharyngodistal myopathy [9, 12, 13, 14, 15, 16, 17, 18, 19, 20, 21, 22, 23, 24, 25]. Expanded repeats are embedded in a small upstream open reading frame, driving the translation of polyglycine (polyG) proteins that accumulate in the nucleus [26, 27, 28, 29, 30]. Consistently, polyG aggregates were detected within the intranuclear inclusions in patient tissues (e.g., brain, skin and muscle) using anti‐uN2CpolyG antibodies [26, 27, 28, 29, 62]. Although polyG is established as a core proteinaceous component of the intranuclear inclusions in NIID, its molecular architecture and higher‐order organization in cells remain poorly understood.

To date, ultrastructural analyses of NIID patient material have largely been limited to conventional electron microscopy, which confirmed a fibrillar appearance but lacked the resolution to define supramolecular organization [2, 3, 7, 31, 32]. A central unanswered question is therefore what biophysical principles drive intranuclear inclusion formation in NIID. In other neurodegenerative disorders, pathogenic aggregates typically form highly insoluble cross‐β amyloid fibrils, and cryo‐electron microscopy (cryo‐EM) has resolved near‐atomic structures of such fibrils purified from patient tissue [33, 34, 35, 36, 37, 38, 39, 40, 41]. PolyG, however, is a biophysical outlier: as a homopolymeric glycine chain, it lacks hydrophobic side chains, aromatic residues, and the intrinsic β‐sheet propensity that characterize many amyloidogenic proteins [42, 43]. How polyG assembles into fibrils and higher‐order inclusion architectures—and which cellular factors participate in or are recruited during this process—remains unknown. Whether polyG assemblies adopt canonical amyloid folds, form branched networks, or exhibit heterogeneous polymorphs in situ has not been systematically addressed, yet resolving this structural logic is essential for understanding polyG‐driven NIID pathology. Emerging studies have implicated polyG aggregates in mitochondrial impairment, disruption of nucleocytoplasmic transport, RNA splicing dysregulation, induction of nucleolar stress, and interference with ribosome biogenesis and stress granule dynamics [27, 28, 29, 44, 45, 46, 47, 48]. However, these observations have largely remained correlative, and it remains unclear whether polyG inclusions exert toxicity through a shared structural mechanism or via sequestration of specific quality‐control factors.

In this work, we integrated disease modeling, cryo‐electron tomography, and in situ proximity proteomics. We generated a Nestin‐NIID transgenic mouse model that recapitulates hallmark NIID pathology, from intranuclear p62‐positive polyG inclusions to ultrastructural features indistinguishable from patient tissue. Using this model, we purified polyG assemblies and applied cryo‐electron tomography (cryo‐ET) to uncover a hierarchical assembly pathway in which ∼5 nm branched fibrils laterally coalesce into compact ribbons. By developing a tracer‐guided cryo‐correlative workflow that enables molecular targeting of polyG inclusions directly within vitrified diseased brain tissue, we show that these ribbon‐shaped architectures represent the dominant in situ conformation. Proximity‐dependent biotinylation further revealed selective enrichment of proteasome complexes and molecular chaperones at polyG ribbons, and cryo‐ET directly visualized proteasome‐like particles engaging ribbon‐shaped surfaces and edges. Collectively, our work defines the native molecular blueprint of NIID inclusions and provides a structural framework for how inclusion architecture can engage the local nuclear proteostasis environment.

## 2 Results

### 2.1 A Transgenic Mouse Model Recapitulates Intranuclear polyG Aggregation in NIID

To delineate the structural properties of polyG aggregates, we generated a Nestin‐NIID mouse model by crossing the conditional transgenic mouse model [28] (carrying the 5′UTR of *NOTCH2NLC* with 98 GGC repeats, with polyG proteins tagged with a 3×Flag epitope) with Nestin‐Cre mice, enabling widespread brain expression of the polyG protein. This model developed widespread polyG inclusions, notable neuronal loss, extensive gliosis, and motor deficits, consistent with our earlier findings in EIIa‐NIID mice [28]. Nestin‐NIID mice were selected because their extended survival (∼P90 vs. ∼P60 for EIIa‐NIID mice) allows structural characterization of intranuclear inclusions at a more mature adult stage.

Immunostaining of Nestin‐NIID mice revealed abundant intranuclear polyG inclusions that colocalized with the autophagy adaptor p62 (Figure 1B,C), closely resembling the pathological hallmark observed in NIID patient tissues (Figure 1A). High‐resolution confocal imaging further revealed morphological heterogeneity: most inclusions appeared as dense intranuclear spheroids, whereas a subset displayed ring‐like morphologies consistent with peripheral enrichment around the nucleoplasm (Figure 1D). Conventional transmission electron microscopy (TEM) of inclusions from both mouse brain tissue and patient skin samples showed comparable ultrastructural features, including electron‐dense cores with more fibrillar material at the periphery (Figure 1E). Together, these observations establish that our mouse model reproduces key immunopathological and ultrastructural characteristics of human NIID inclusions.

**FIGURE 1:**
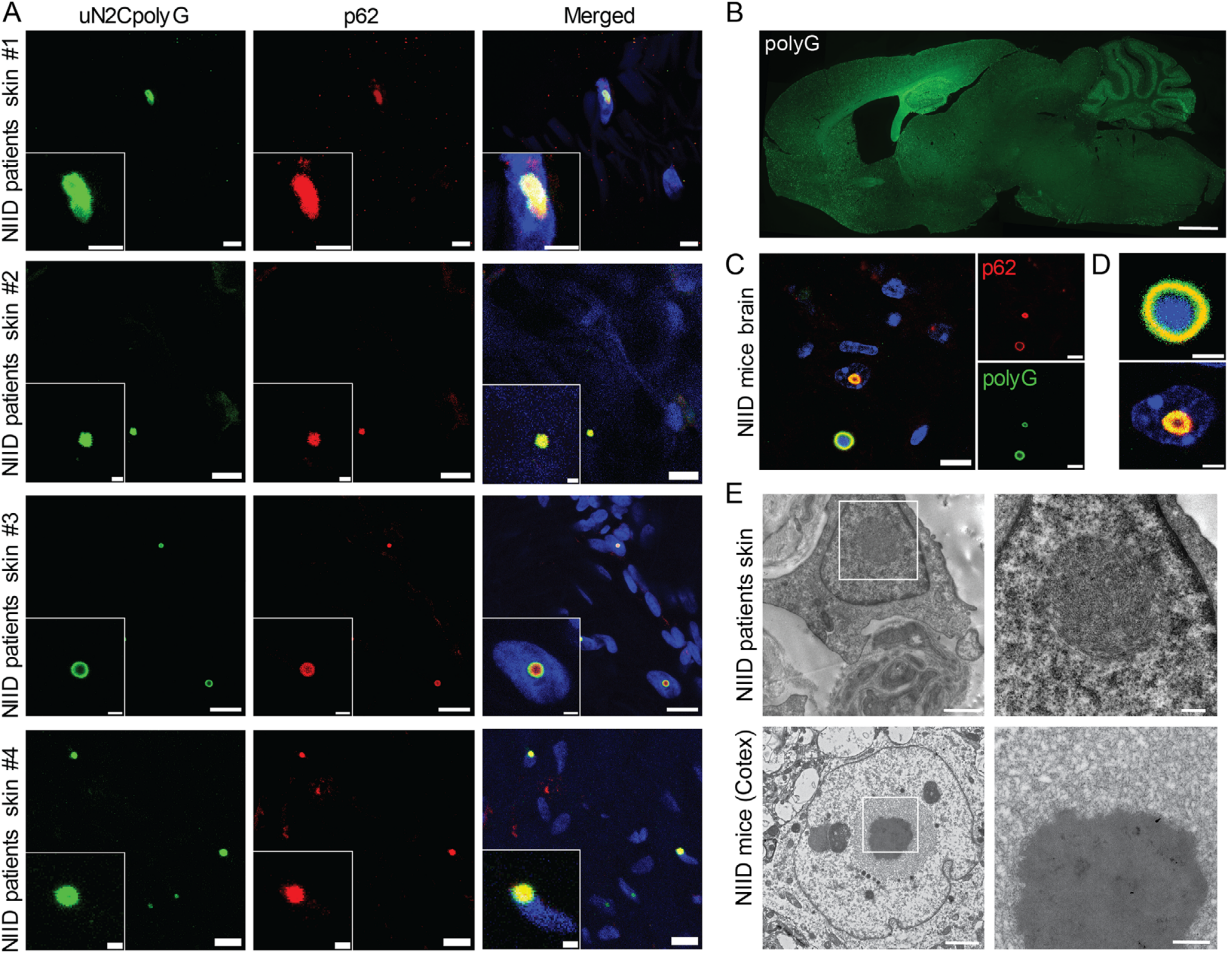
A *NOTCH2NLC* GGC‐expansion mouse model recapitulates intranuclear polyG inclusions with NIID‐like histopathological hallmarks. (A) Immunofluorescence images of skin biopsies from four patients with NIID, stained for polyG (green) and p62 (red). Nuclei are counterstained with DAPI (blue). Scale bar: 10 µm (overview), 3 µm (inset). (B) Immunofluorescence staining of a whole‐brain section from an NIID mouse model using anti‐Flag antibody (green). Scale bar: 1 mm. (C) High‐magnification confocal images of the mouse hippocampus showing colocalization of polyG (anti‐Flag, green) with p62 (red) in intranuclear aggregates. Nuclei are labeled with DAPI (blue). Scale bar: 10 µm. (D) Morphological diversity of polyG aggregates in mouse neurons: dense compact aggregates (top) and ring‐like aggregates (bottom). Anti‐Flag (green); Anti‐p62 (red); DAPI (blue), Scale bar: 3 µm. (E) Conventional transmission electron microscopy (TEM) images of intranuclear inclusions from an NIID mouse model cortex (bottom) and a skin fibroblast from an NIID patient (top). Fibrillar structures are visible within electron‐dense inclusions. Scale bar: 2 µm (overview), 500 nm (inset).

### 2.2 Brain‐Extracted polyG Aggregates Form Heterogeneous Fibrillar and Ribbon‐Shaped Assemblies

To define the nanoscale architecture of the polyG inclusion core, we isolated and purified polyG assemblies from NIID mouse brains (Figure 2A and Figure S1A). Western blot and negative‐stain EM combined with immunogold labeling confirmed enrichment of polyG‐positive fibrillar material, along with broader ribbon‐like structures suggestive of higher‐order organization (Figure 2B and Figure S1B–D).

**FIGURE 2:**
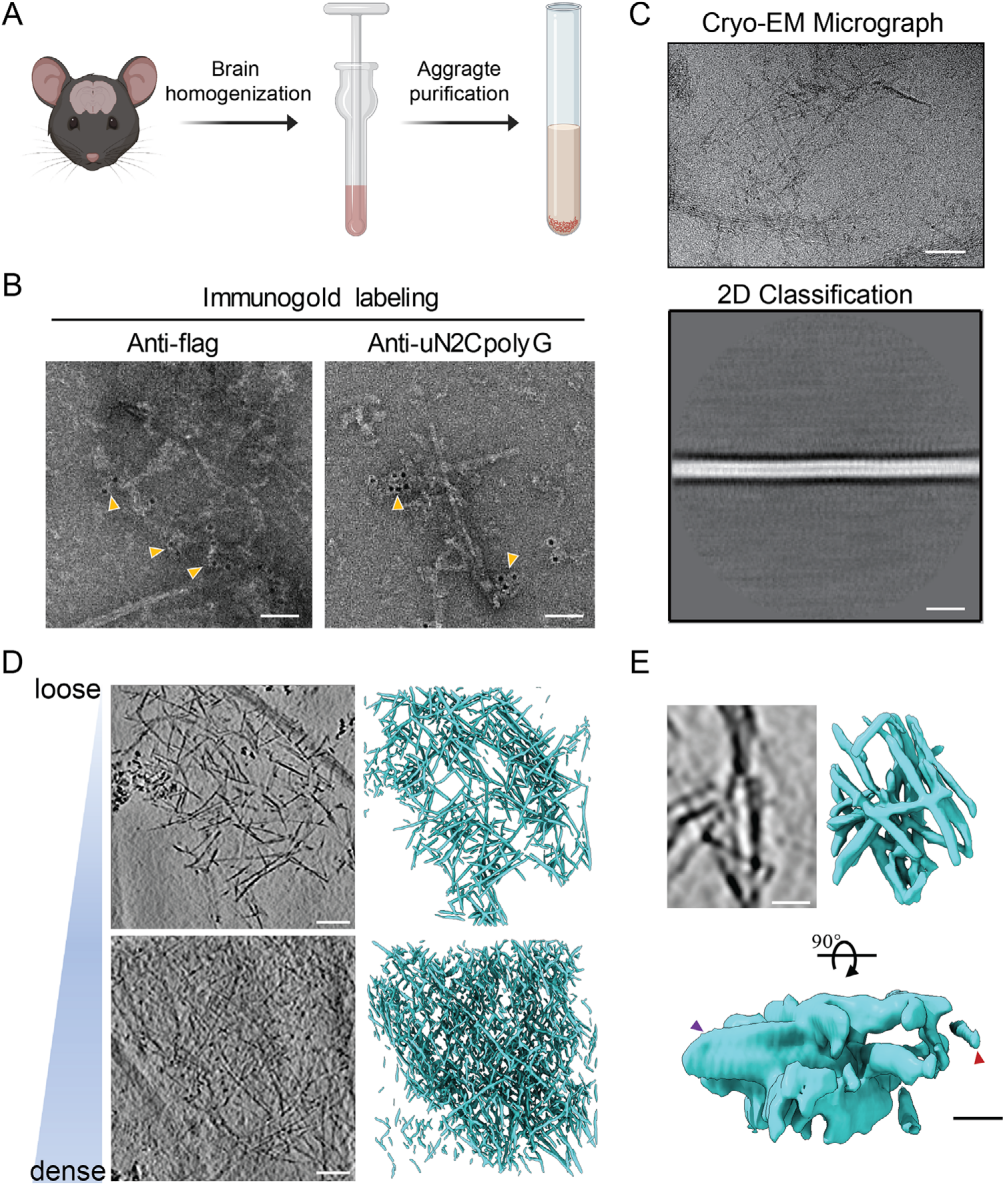
Brain‐extracted polyG aggregates form heterogeneous fibrillar and ribbon‐shaped assemblies. (A) Schematic workflow for purifying polyG aggregates from NIID mouse brain tissue. (B) Negative‐stain TEM of purified polyG aggregates. Immunogold labeling with anti‐Flag (left, 6‐nm gold particles) and anti‐polyG tag (right, 6‐nm nanogold particles) confirms the fibrillar structures as polyG aggregates. Scale bar: 50 nm. (C) Cryo‐electron microscopy (cryo‐EM) analysis of polyG aggregates. Top: Representative cryo‐EM micrograph. Bottom: Representative 2D class averages from single‐particle analysis. Scale bar: 50 nm (micrograph), 10 nm (2D class). (D) Cryo‐electron tomography (cryo‐ET) of purified polyG aggregates. Montage of tomographic slices (left) and corresponding 3D segmentations (right) reveal a structural continuum from loose fibrillar networks to densely packed aggregates. Scale bar: 100 nm. (E) Higher‐magnification views and topological analysis of polyG fibrils. Enlarged views show coexistence of fibrils (red arrowheads) and ribbon‐like structures (purple arrowheads). Note frequent branching, crossing, and lateral fibril associations that drive ribbon formation. Scale bar: 20 nm.

We next performed cryo‐EM imaging on the brain‐extract samples. While fibrillar features were readily apparent, the particles displayed extensive heterogeneity, branching, and lateral association, which limited the ability to obtain interpretable class averages with a consistent axial repeat or to perform conventional helical reconstruction (Figure 2C and Figure S3A). The power spectrum of the electron micrographs and Fourier transforms of 2D‐averaged density revealed a weak but detectable signal at approximately 4.7 Å, consistent with cross‐β‐like inter‐strand spacing. However, this signal was not accompanied by a robust helical layer‐line pattern across the dataset (Figure S3B,C). These observations indicate that purified polyG aggregates contain amyloid‐like fibrillar components, but are not dominated by a single rigid, canonical helical amyloid filament. This structural heterogeneity motivated the use of cryo‐ET to resolve three‐dimensional organization without imposing helical symmetry or strict periodicity constraints.

Cryo‐ET reconstructions revealed a continuum of architectures ranging from loosely connected fibrillar networks to highly compact assemblies (Figure 2D). At higher magnification, we observed that the fundamental building block is a ∼5 nm fibril that frequently branches and intersects (Figure 2E and Figure S3 and Movie S1). These fibrils also engage in extensive lateral interactions, aligning in parallel and packing tightly to form sheet‐like, ribbon‐shaped structures. Quantitative analysis of purified polyG aggregates from mouse brain further demonstrated that ribbon‐shaped assemblies account for over 80% of all identifiable aggregates, whereas loosely organized fibrils and clustered fibrils represent a minor fraction (Figure S4). This mode of organization is relevant to classical structural studies of polyglycine, which described two major conformational states: polyglycine‐I, characterized by antiparallel β‐sheet‐like packing, and polyglycine‐II, an extended 3_1_‐helical conformation that can assemble into ordered arrays [49, 50, 51]. Although the resolution of our tomograms does not allow assignment of the polyG ribbons to either conformation, these earlier studies support the idea that glycine‐rich chains can adopt extended, backbone‐dominated conformations with diverse packing modes. Quantification of fibril diameters and ribbon‐shaped widths supported this hierarchical “fibril‐to‐ribbons” assembly model (Figure S3). Thus, purified polyG assemblies are not dominated by a single rigid filament but instead comprise branched ∼5 nm fibrils that laterally pack into higher‐order ribbons, consistent with a higher‐order organization of laterally packed polyglycine‐rich fibrillar units.

### 2.3 In Situ Molecular Tracing Visualizes Ribbon‐Shaped polyG Assemblies Within Neuronal Nuclei

Recent technological advances in cryo‐focused ion beam (FIB) milling and cryo‐ET have enabled the visualization of unperturbed subcellular architecture [52, 53, 54, 55] and have now extended to thick tissue samples [56, 57, 58, 59], permitting investigation of protein aggregates *in tissue*. To establish the physiological organization of polyG inclusions, we optimized an integrated workflow combining molecular tracing with in situ cryo‐ET for thick brain tissue, adapted from previously described methodologies [60] (Figure 3A). As an operational tracer for targeting inclusions, we tested the amyloid‐binding dye Thioflavin S (ThS) and observed substantial colocalization with polyG‐positive inclusions in NIID mouse brain sections (Figure 3B). Because polyG lacks several sequence features typical of classical amyloid‐forming proteins, we further tested additional amyloid‐binding tracer in NIID mouse brain sections (Figure S5A,B). These probes labeled polyG‐positive inclusions to varying extents, supporting the presence of amyloid‐like components within the aggregates. This interpretation is consistent with the weak ∼4.7 Å signal detected in Fourier transforms of purified polyG assemblies. However, the heterogeneous staining patterns and the lack of a dominant helical fibril architecture indicate that polyG inclusions are distinct from canonical amyloid deposits. Thus, ThS was used here as an operational tracer for targeting inclusion‐rich regions, rather than as definitive evidence of a classical amyloid fold. This chemical probe‐based approach, which does not rely on fluorescent reporter labeling, is readily applicable to more pathologically relevant specimens, including patient‐derived tissues.

**FIGURE 3:**
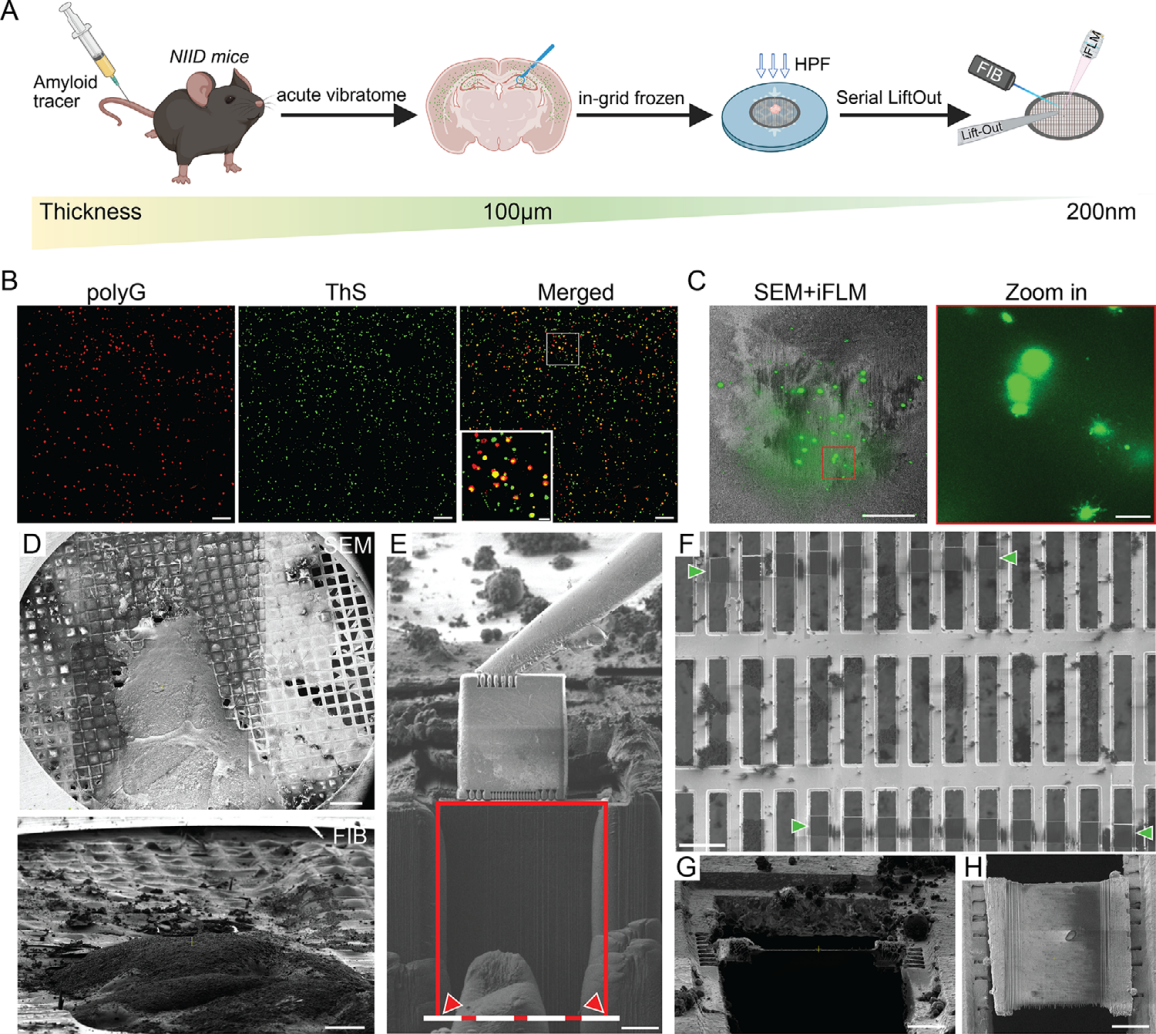
In‐grid high‐pressure freezing coupled with Serial Lift‐Out enables targeted in situ Cryo‐ET of polyG aggregates. (A) Schematic workflow for preparing NIID mouse brain tissue for in situ structure determination by cryo‐CLEM and cryo‐ET. HPF, high‐pressure freezing. (B) Validation of amyloid tracer. Confocal immunofluorescence image of an NIID mouse brain section showing co‐localization of injected Thioflavin S (ThS, green) with polyG aggregates (anti‐Flag, red). Scale bar: 50 µm (overview), 10µm (inset). (C) Cryo‐fluorescence imaging for target identification. Left: Cryo‐FIB/SEM image showing the morphology of vitrified brain tissue with scanning electron microscopy. ThS‐positive signals (green) are overlaid. Right: Higher‐magnification view of a ThS‐positive aggregates (red dashed box). Scale bar: 50 µm (overview), 5µm (inset). (D–H) Cryo‐FIB/SEM‐based lamella preparation. (D) Morphology of vitrified brain tissue imaged by scanning electron microscopy (top) and focused ion beam (bottom). Scale bar: 200 µm. (E) Micromanipulator needle lifting the tissue chunk containing the region of interest, with the red arrow and white line indicating the cutting direction at the lower edge Scale bar: 10 µm (F) Lamella after attached to a TEM grid for subsequent Cryo‐ET tilt‐series acquisition. The green arrow points to the lamellae attached to the grid. Scale bar: 100 µm. (G,H) Focused ion beam (G) and scanning electron (H) images of a lamella after thinning to ∼200 nm. Membrane structures within the tissue, such as myelin sheaths, are discernible. Scale bar: 10 µm.

Based on regional pathological burden, we selected hippocampus for further in situ analyses. NIID mouse model received intravenous ThS (a widely used amyloid dye), and tissue was rapidly processed and vitrified using an in‐grid high‐pressure freezing approach that preserves native ultrastructure in 120–150 µm thick samples (See Methods for details). Frozen grids were transferred to a cryo‐FIB/SEM equipped with cryo‐fluorescence imaging, enabling precise targeting of ThS‐positive inclusion regions within vitrified tissue (Figure 3C). Using an optimized serial lift‐out workflow, targeted regions were extracted, transferred to lift‐out grids, and thinned into ∼200 nm lamellae suitable for cryo‐ET (Figure 3D–H). This pipeline enabled reproducible acquisition of in situ tomograms across multiple samples (Figure S6A).

Cryo‐fluorescence microscopy integrated with FIB/SEM enabled precise localization of polyG inclusions (Figure 4A–C and Figure S6A). In native, unfixed NIID mouse brain tissue, inclusions were clearly delineated within intact nuclear membranes, featuring electron‐dense cores surrounded by diffuse fibrillar structures at the periphery. Cryo‐ET reconstructions further resolved the three‐dimensional organization of polyG assemblies within intact neuronal nuclei (Figure 4D–G). In situ inclusions predominantly adopted compact ribbon‐shaped architectures composed of densely packed, laterally associated fibrillar units (Figure 4H and Figure S6B). Analysis of 33 tomograms from six independent inclusions revealed that ribbon‐shaped assemblies dominate both the peripheral shell‑halo and the core regions, with no other structural states observed. Notably, ribbons in the peripheral halo appeared longer and more loosely organized than their more compact counterparts in the core. Compared with purified material, in situ ribbons were generally more compact with tighter inter‑sheet connectivity, yet the core hierarchical assembly principle of “branched fibrils to ribbons” remained unchanged. These findings are consistent with the structural characteristics of purified polyG assemblies, further demonstrating that ribbon‐shaped architectures represent the dominant morphological state of polyG aggregates.

**FIGURE 4:**
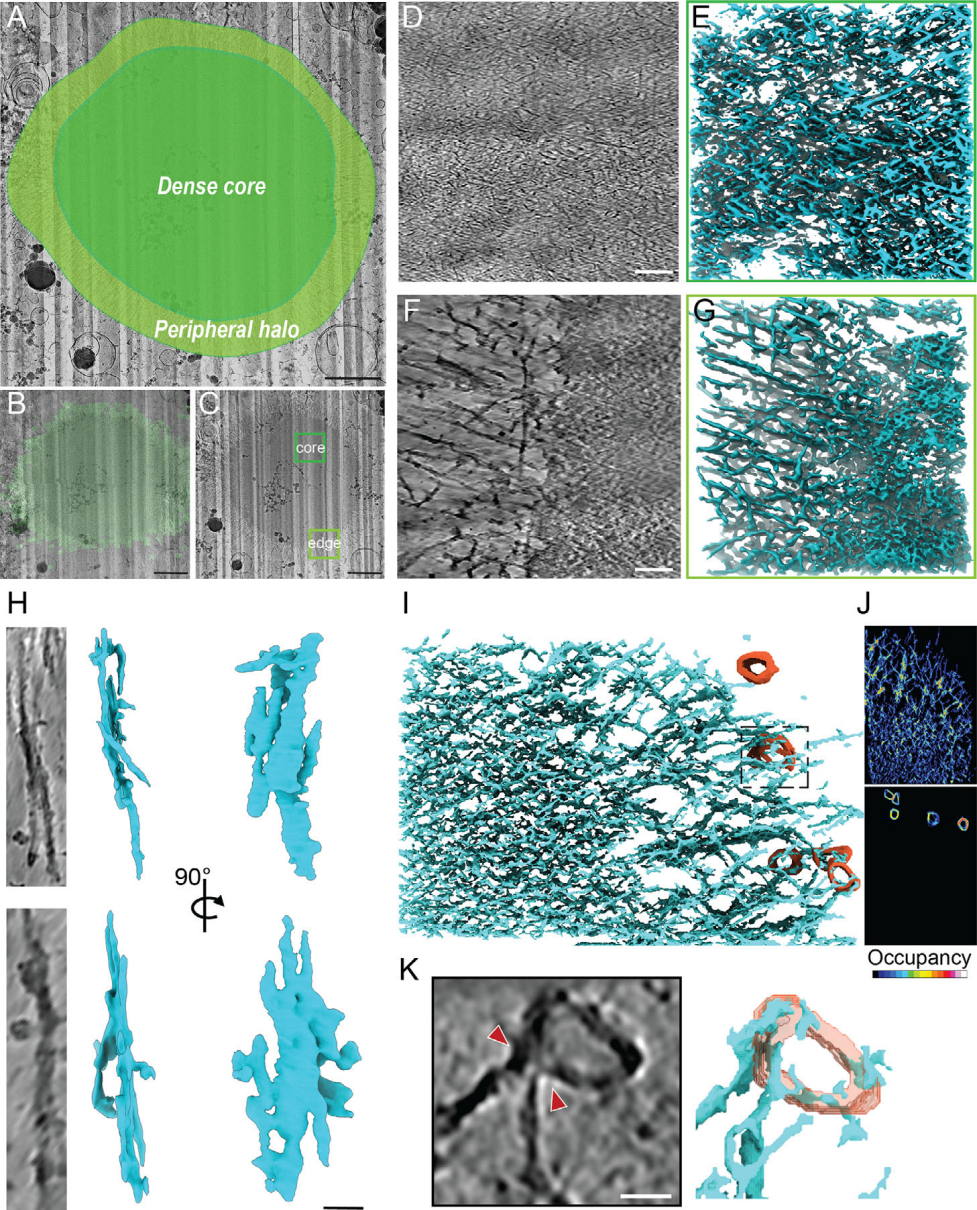
Molecular architecture of polyG inclusions resolved by *in‐tissue* Cryo‐ET. (A) Low‑magnification segmentation of an *in tissue* polyG inclusion within NIID mouse brain, illustrating the electron‑dense core and the surrounding diffuse peripheral halo. (B) Cryo‑correlative light and electron microscopy image corresponding to the region highlighted in (A). (C) Schematic indicating the data acquisition positions for the dense core (dark green box) and peripheral halo (light green box) within the same inclusion. (D) Representative tomographic slice of the dense core. (E) Three‑dimensional segmentation corresponding to (D), showing the organization of polyG aggregates. (F) Representative tomographic slice of the peripheral halo region. (G) Three‑dimensional segmentation corresponding to (F). (H) Selected ribbons from (F) rotated and magnified for visualization. (I) Three‑dimensional segmentation of a polyG inclusion showing the spatial distribution of polyG ribbons (Cyan) and cellular membrane components (magenta). (J) Maximum intensity projection heatmaps shown in (I), highlighting the spatial distribution of polyG aggregates and cellular membrane components. (K) Magnified view of the boxed region in (I), revealing direct contact (arrowheads) between membrane vesicles and polyG ribbons. Scale bar: 500 nm (A–C); 50 nm (D–G); 20 nm (H); 25 nm (K).

Recent super‐resolution fluorescence microscopy studies have reported that polyG inclusions assemble into ordered core‐shell structures [61]. Consistent with this, pathological polyG inclusions in native NIID mouse brain exhibited a clear core‐periphery boundary. In the core region, polyG ribbons formed densely interwoven networks with sparse membranous structures. In contrast, the periphery displayed a transition from compact ribbons to diffuse fibrillar material, accompanied by relatively enriched membrane components. Three‐dimensional reconstructions of the inclusion core (Figure 4D,E) and periphery (Figure 4F,G) revealed heterogeneous distribution of these ribbon‐shaped structures, which may explain the layer‐specific co‐deposition of distinct protein markers. In addition, three‐dimensional segmentation further revealed close spatial proximity between ribbons and surrounding nuclear/perinuclear membranous structures. Direct contact between polyG ribbons and membrane components induced membrane remodeling and deformation (Figure 4I–K and Figure S7). Together, these findings support that the ribbon‐shaped scaffold represents the predominant physiological architecture of polyG inclusions and polyG ribbons impinge on cellular endomembranes in NIID brain tissue.

### 2.4 Proximity Proteomics Identifies Proteostasis Machinery Enriched Around polyG Inclusions

To systematically decipher the molecular interaction network of polyG aggregates within cells, we employed a previously reported in situ proximity‐dependent proteomics technique based on horseradish peroxidase (HRP)‐catalyzed biotinylation [62]. This method enables specific biotinylation and affinity enrichment of proteins proximal to polyG aggregates directly in fixed brain tissue sections (Figure 5A). Immunofluorescence confirmed strong overlap between polyG inclusions and streptavidin signal (Figure 5B), consistent with selective labeling of proteins near inclusions, whereas control tissue showed minimal background labeling (Figure S8).

**FIGURE 5:**
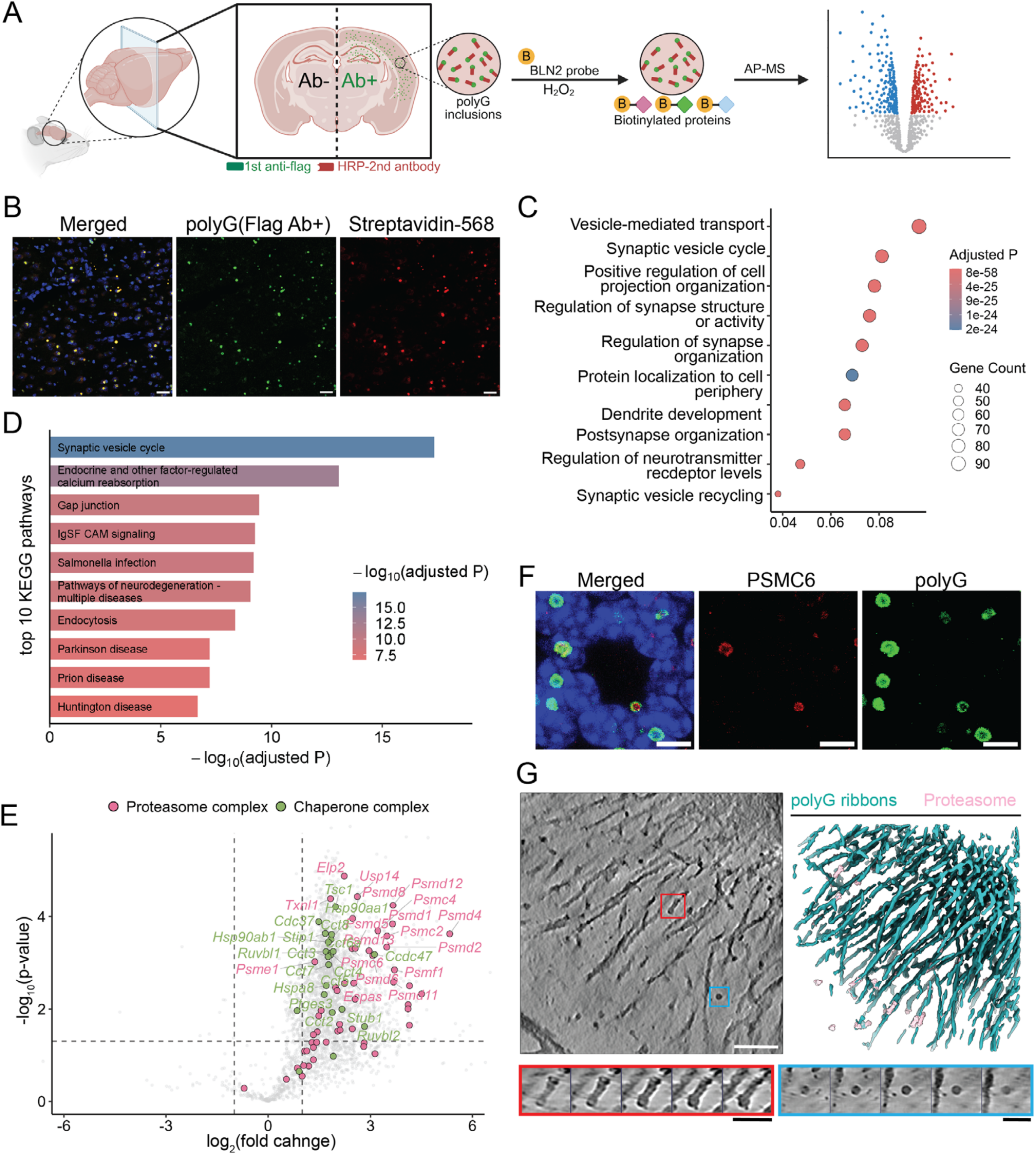
Proximity proteomics identifies proteostasis machinery enriched around polyG inclusions. (A) Schematic of the HRP‐based in situ proximity labeling workflow. NIID mouse brain sections were sequentially incubated with anti‐Flag primary antibody, HRP‐conjugated secondary antibody, and multi‐functional BLN2 probe, enabling both fluorescence validation and subsequent enrichment of biotinylated proteins for mass spectrometry analysis.(B) Specificity validation of the inclusion‐proximal protein biotinylated. Confocal images of NIID mouse brain sections after proximity labeling show colocalization of polyG aggregates (anti‐Flag, green) with biotinylated proteins (streptavidin, red). Nuclei are counterstained with DAPI (blue). Scale bar: 25 µm. (C) Gene Ontology Biological Process (GO‐BP) analysis of proteins significantly enriched in the polyG‐proximal proteome. (D) KEGG pathway analysis. Bar graph showing the top enriched pathways, revealing a strong association with various neurodegenerative diseases. (E) Volcano plot of the polyG‐proximal proteome, highlighting significant enrichment of protein quality control components. Proteasome subunits are shown in magenta, and chaperone components are shown in green. (F) Immunofluorescence image showing colocalization of a proteasome subunit (PSMC6, red) with polyG aggregates (anti‐Flag, green) in an NIID mouse brain section. Scale bar: 5 µm. (G) Representative cryo‑ET tomogram (left) and its corresponding 3D segmentation (right) showing enrichment of proteasome‑like complex within polyG inclusion. PolyG ribbons are colored in cyan, and proteasome‑like particles are shown in pink. Bottom: series of higher‑magnification tomographic slices of representative proteasome complexes detected in the tomogram are shown. Scale bar: 50 nm (tomogram), 25 nm (the multi‑Z slices). See also Movie S2 and Table S1.

Proteomic analysis of biotinylated proteins identified an inclusion‐proximal interactome enriched for neuronal projection, protein localization and trafficking‐related annotations by Gene Ontology (GO) analysis (Figure 5C and Table S1), consistent with an altered molecular environment in the vicinity of inclusions. Furthermore, KEGG pathway analysis revealed polyG‐associated proteins significantly enriched in multiple neurodegeneration‐associated pathways, including Parkinson's, Huntington's, and prion disease (Figure 5D), potentially explaining the heterogeneous clinical manifestations of NIID (Figure 5D). Several previously reported polyG interactors—including SQSTM1, UBB [26, 27, 29, 61, 63], LRPPRC [29], HNRNPM [28], FAM98B, RCTB, DDX1, and RTRAF [45], TAX1BP1, and TOLLIP [47], as well as FUS [61]—were significantly enriched in our proximity proteomics dataset, whereas KU70/80 [26], NFΚB p65 [46], HSPB1 [47], and PML [61] was not detected above background under our experimental conditions (Figure S10). These findings independently validate a core subset of proposed interactions and suggest that some associations may be cell‐type, species, or aggregation‐state dependent. Notably, multiple proteostasis components were also strongly enriched. Volcano plot and intersection analyses highlighted significant enrichment of proteasome subunits and molecular chaperones, including HSP90 family members (Figure 5E). Immunofluorescence further validated colocalization of a proteasome subunit with polyG inclusions not only in the nuclei but also in extranuclear regions, as observed in both NIID mouse brain and NIID patient skin (Figure 5F and Figure S9).

Notably, revisiting our in situ cryo‐ET datasets, we observed macromolecules in the inter‐ribbon spaces that appeared as ∼10‐nm rings in tomographic cross‐sections from five independent inclusions. This finding indicates that proteasome recruitment is a general characteristic of polyG inclusions. Positioned in close apposition to or direct contact with polyG ribbons, these particles matched the size and morphology of proteasome complexes, exhibiting characteristic ring‑shaped or barrel‑like architectures with a central cavity. However, the limited number of proteasome‐like particles currently precludes subtomogram averaging to further resolve their conformation. Of note, a recently reported deep learning‐based denoising approach enables direct determination of cellular structures at submolecular resolution without averaging. Applying this segmentation methods, we were able to directly render the densities of proteasome‐like particles within polyG inclusions, which closely resemble previously reported high‐resolution structures of the 26S proteasome (Figure 5G and Figure S11 and Movie S2). Together, proximity proteomics and cryo‐ET provide orthogonal evidence that all recruit chaperones and proteasome components. We propose that the extensive, high‐valency interfaces presented by dense compact ribbons can recruit proteasomes and chaperones—potentially reflecting an attempted clearance response—while persistent association could also sequester or stall quality‐control components, thereby reducing effective nuclear proteolytic capacity and amplifying proteome imbalance in NIID.

## 3 Discussion

In this work, we characterize the nanoscale architecture and local molecular environment of polyG inclusions in NIID, providing structural context for how intranuclear inclusions may influence nuclear homeostasis. By integrating cryo‐ET of brain‐extracted aggregates with tracer‐guided *in tissue* cryo‐ET, we show that polyG inclusions predominantly form compact ribbon‐shaped scaffolds built from branched ∼5 nm fibrillar units that laterally associate into densely packed sheets. This organization lacks the pronounced axial periodicity and consistent helical symmetry characteristic of many canonical amyloid fibrils [33, 34, 35, 36, 37, 38, 39, 40, 41]. The agreement between brain‐extracted and in situ reconstructions supports the conclusion that ribbon‐shaped packing is a bona fide organizational state of polyG assemblies rather than a byproduct of biochemical isolation. In parallel, our in‐tissue cryo‐ET workflow—combining tracer‐enabled cryo‐fluorescence targeting, in‐grid high‐pressure freezing, and an optimized serial lift‐out procedure—enables systematic tomographic analysis of intranuclear inclusions in thick brain tissue.

These results suggest that polyG aggregation follows a distinct supramolecular assembly principle. Given the low‐complexity sequence of polyG, our data support a hierarchical model in which flexible fibrils engage in multivalent branching and extensive lateral packing to generate higher‐order ribbons. This framework provides a plausible structural explanation for the morphological plasticity observed by confocal microscopy, including dense spheroids and ring‐like inclusions, which may reflect different maturation states, spatial constraints, or local nuclear environments. The core‐periphery appearance of polyG inclusions also invites comparison with Aβ plaques. Recent in‐tissue cryo‐ET studies have shown that Aβ plaques are dominated by classical amyloid fibrils and protofilaments arranged in parallel arrays or lattice‐like networks, along with non‐amyloid components such as extracellular vesicles, lipid droplets, and multilamellar bodies [64, 65]. In contrast, although fluorescence microscopy and conventional TEM revealed dense polyG inclusion cores with more fibrillar peripheral material and occasional ring‐like morphologies, our in situ cryo‐ET did not show a separate ring‐like or spherical shell architecture. Instead, polyG inclusions were predominantly characterized by ribbon‐shaped assemblies that are highly compressed and dense at the center, with a more dispersed and unevenly packed periphery; few vesicle‐like components were detected within the inclusion core. Thus, this distinction may reflect their distinct environments: extracellular, membrane‐rich neuropil for Aβ plaques vs. the macromolecule‐dense nuclear compartment for polyG inclusions. Determining the molecular determinants that promote branching and ribbon‐shaped packing—potentially influenced by nuclear crowding, post‐translational modifications, or co‐assembling factors—will be important for connecting sequence‐level constraints to the observed ultrastructure.

These observations also position polyG inclusions within the broader landscape of repeat‐derived protein assemblies. Earlier electron microscopy studies described NIID inclusions as fibrillar networks [2, 3, 7, 31, 32], whereas our in situ cryo‐ET resolves them as compact ribbons formed by laterally packed fibrillar units. This architecture contrasts with the more uniform amyloid‐like filaments formed by polyQ‐expanded huntingtin exon 1 in mammalian cells [66, 67], but resembles the ribbon‐like polyGA assemblies recently observed in C9ORF72 ALS/FTD neuronal models [68]. The structural similarity between polyG and polyGA, both glycine‐rich low‐complexity sequences, suggests that limited side‐chain bulk may promote lateral packing rather than isolated helical fibril formation. Despite these architectural differences, polyQ, polyGA, and polyG inclusions have all been reported to associate with chaperones and proteasome components [68, 69]. By directly visualizing this proteostasis response on polyG ribbons in diseased brain, suggesting that repeat‐expansion inclusions may converge mechanistically through high‐valency aggregate surfaces that sequester or stall quality‐control factors and chronically perturb nuclear protein homeostasis.

A notable feature of in situ assemblies is their increased compaction and interconnection relative to purified material, implying that the nuclear milieu promotes higher‐order connectivity. Such compaction may be functionally consequential: dense ribbon‐shaped scaffolds could occupy nuclear volume, reshape local macromolecular organization, and influence nuclear integrity and transport. While our current datasets do not directly resolve interactions with nuclear pores or the nuclear envelope, the proximity of ribbons to nuclear and perinuclear membranous elements highlights nucleocytoplasmic transport and nuclear architecture as concrete downstream axes for future mechanistic interrogation.

A central mechanistic insight is that polyG ribbons selectively concentrate proteostasis machinery. In situ proximity proteomics identifies enrichment of proteasome subunits and molecular chaperones near inclusions, and cryo‐ET provides structural support by visualizing proteasome‐like particles associated with ribbon‐shaped surfaces and edges. Together, these orthogonal lines of evidence indicate that NIID inclusions are sites of focused quality‐control engagement. We propose that ribbon‐shaped scaffolds, by presenting extended high‐valency interfaces, can recruit proteasomes and chaperones; this may reflect an attempted clearance response, but persistent association could also sequester or stall proteostasis components, reducing effective nuclear proteolytic capacity and amplifying proteome imbalance.

Long‐read sequencing has recently facilitated the identification of an expanding spectrum of neuromuscular disorders caused by GGC repeat expansions in multiple distinct genes [8, 70, 71, 72, 73, 74]. Notably, these GGC repeats, which are predominantly located in non‐coding regions, drive the production of polyG proteins that accumulate to form intranuclear inclusions central to disease pathogenesis [28, 30, 47, 70, 75]. In this study, we combined biochemical purification with in situ electron microscopy to directly visualize intratissue polyG aggregates and for the first time, characterize the non‐canonical fibrillar structure of polyG produced from *NOTCH2NLC* GGC repeat expansions. Our integrated strategy establishes a methodological framework for future investigations into the assembly of distinct polyG aggregates and their impacts on cellular function.

This study has limitations. Although the transgenic mouse model robustly recapitulates hallmark pathology of human patients, Nestin‐driven expression of polyG differs from the endogenous, age‐dependent accumulation observed in patients. This difference in spatiotemporal dynamics, together with the multisystem nature of NIID, may influence inclusion kinetics, composition, or maturation. Thus, the ribbon architecture and associated molecular interactions defined here were characterized in this model system; their relevance to human patient inclusions is strongly suggested by pathological concordance, but remains to be validated in human post‐mortem tissue using comparable in situ cryo‐ET workflows. Additionally, although ThS‐enabled targeting is operationally effective and supported by colocalization, it does not establish chemical specificity by itself. Proximity biotinylation reports spatial proximity rather than direct binding, highlighting the need for functional perturbations to establish causality. Similarly, although our proximity proteomics and cryo‐ET data show that proteasome subunits and molecular chaperones are enriched around polyG ribbons, these observations establish spatial association rather than functional causality. Thus, the proposal that persistent recruitment of proteostasis machinery imposes a burden on nuclear protein homeostasis remains a mechanistic hypothesis. Functional studies measuring proteasome and chaperone activity, together with genetic or pharmacological perturbation of these pathways, will be required to determine whether their recruitment to polyG ribbons directly contributes to polyG toxicity, impaired aggregate clearance, or nuclear proteostasis dysfunction. Despite these constraints, our work establishes an architecture‐to‐mechanism framework in which branched fibrils coalesce into compact nuclear ribbons that concentrate proteostasis machinery. Future studies mapping architectural states across disease progression, validating them in human tissue, and perturbing proteostasis pathways will help determine whether targeting the ribbon‐shaped scaffold—or its proteostasis interfaces—can mitigate NIID‐associated nuclear dysfunction.

## 4 Experimental Section

### 4.1 Human Samples

Participants were recruited from Xiangya Hospital, Central South University. Each case was independently evaluated by at least two neurologists. The diagnosis of NIID was established based on previously published criteria [9]. All human skin samples used for immunofluorescence imaging and electron microscopy were obtained with written informed consent from participants. This study was approved by the Ethics Committee of Xiangya Hospital, Central South University, China (Approval No. 2024040420).

### 4.2 Laboratory Animals

The Nestin‐NIID mouse model was generated by crossing a conditional transgenic line [28] carrying 5′UTR of *NOTCH2NLC* with 98 GGC repeats—preceded by a loxP stop loxP cassette—with Nestin‐Cre mice (J003771, from JAX), enabling widespread polyG expression throughout the brain. No obvious sex‐dependent differences in neuropathology or behavioral phenotypes were observed. P75 Nestin‐NIID mice and littermate controls (conditional transgenic mice without Cre expression) were used for tissue collection, histological analysis, biochemical extraction, and cryo‐ET experiments, regardless of gender. All animal procedures were approved by the Animal Care and Use Committee of Central South University (Approval No. 202309022), and mice were bred and maintained on a 12:12 h light/dark cycle under specific pathogen‐free conditions in the animal facility of Xiangya Hospital, Central South University.

### 4.3 Immunofluorescent Staining

#### 4.3.1 Human Skin Samples

Tissues were fixed overnight in 4% PFA at 4°C, cryoprotected in graded sucrose solutions (20%–30% w/v in PBS), embedded in OCT, and sectioned at 30 µm using a cryostat (Leica CM3050 S). Sections were incubated with primary antibodies overnight at 4°C, washed with PBST, and incubated with secondary antibodies for 2 h at room temperature. Nuclei were stained with DAPI. Autofluorescence was quenched with 3% Sudan Black B. Sections were mounted with ProLong Gold Antifade Mountant.

#### 4.3.2 Mouse Brain Slices

Mice were perfused transcardially with PBS and 4% PFA. Brains were post‐fixed, cryoprotected in sucrose, and cut into 30 µm coronal sections using a vibratome. Free‐floating sections were blocked (5% BSA, 0.2% Triton X‐100 in PBS) for 1 h, incubated with primary antibodies overnight at 4°C, washed, and incubated with secondary antibodies for 2 h. Nuclei were stained with DAPI. For THS co‐staining, sections were incubated with 10 µM Thioflavin S in 30% ethanol for 2 h prior to blocking. Sections were mounted with ProLong Gold Antifade Mountant.

#### 4.3.3 Antibodies

The following primary antibodies were used: rabbit polyG antibody PEP122 (developed in‐house in a previous study) [28], anti‐SQSTM1/p62 mouse monoclonal antibody (1:500; Abcam, ab56416), anti‐PSMC6 mouse monoclonal antibody (1:1000; Abcam, ab22639) and anti‐FLAG M2 antibody (1:500; Cell Signaling Technology, 14793). The secondary antibodies used were: goat anti‐rabbit IgG (H+L) cross‐adsorbed, Alexa Fluor 488 (1:1000; Invitrogen, A‐11008) and goat anti‐mouse IgG (H+L) cross‐adsorbed, Alexa Fluor 568 (1:1000; Invitrogen, A‐11004).

#### 4.3.4 Imaging

Fluorescent images were acquired using a confocal microscope (Leica SP8). Imaging parameters for individual channels, including laser intensity and exposure time, were kept consistent throughout the experiment. A 20 × air objective was used for image acquisition. All fluorescent images were analyzed using ImageJ software.

### 4.4 Room‐Temperature Electron Microscopy

Human skin and mouse brain samples were processed identically for room‐temperature TEM. Tissues were trimmed to 1 × 1 × 3 mm^3^, fixed in 2.5% glutaraldehyde (in Millonig's phosphate buffer, pH 7.3), post‐fixed with 1% osmium tetroxide, dehydrated in graded acetone, and resin‐embedded. Ultrathin sections (70–100 nm) were cut with a diamond knife on an ultramicrotome (Leica Microsystems), double‐stained with 3% uranyl acetate and lead citrate, and imaged on a Tecnai T12 microscope (FEI) operating at 120 kV.

### 4.5 Ex Vivo Purification

Extraction of aggregates from frozen brain tissues of NIID mice was performed based on a previously described method with modifications [76]. Briefly, frozen mouse brain tissue (0.35 g) was homogenized in 40 volumes (v/w) of extraction buffer containing 10 mM Tris‐HCl (pH 7.5), 0.8 M NaCl, 10% sucrose, and 1 mM EGTA. The homogenate was adjusted to 2% sarkosyl and incubated at 37°C for 30 min with constant agitation, followed by centrifugation at 2000 × g for 10 min at 25°C. The supernatant was subjected to ultracentrifugation at 166 000 × g for 20 min at 25°C using an SW41Ti rotor (Beckman Coulter), yielding sarkosyl‐soluble and sarkosyl‐insoluble fractions. The sarkosyl‐insoluble pellet was resuspended by sonication (Titec VP‐5s, power setting 2, 1 sec/pulse, 5 pulses) in extraction buffer supplemented with 1% sarkosyl (1 mL per gram of original tissue). The resuspended material was diluted fourfold with the same buffer, incubated at 37°C for 20 min, and centrifuged at 2,000 × g for 15 min at 25°C. The resulting supernatant was then centrifuged at 166 000 × g for 30 min. The final sarkosyl‐insoluble pellet was resuspended by sonication (same parameters as above) in 20 mM Tris‐HCl (pH 7.4) and 150 mM NaCl for subsequent electron microscopy characterization.

### 4.6 NS‐TEM and Immunogold NS‐TEM

For conventional negative‐staining TEM, an aliquot of 3 µL of mouse brain‐extracted sample was applied to glow‐discharged 200‐mesh carbon‐coated copper grids (Beijing Zhongjingkeyi Technology). After a 45 s incubation, excess sample was blotted away with filter paper. The grids were then sequentially washed with 5 µL of double‐distilled H_2_O and 5 µL of 2% (w/v) uranyl acetate, followed by staining with an additional 5 µL of 2% (w/v) uranyl acetate for 45 s. After air‐drying, the grids were examined using a Tecnai T12 microscope (FEI) operated at 120 kV.

For immunogold labeling NS‐TEM, 5 µL of sarkosyl‐soluble brain‐extracted fibrils incubated with 0.4 mg/mL pronase (Roche) for 15 min at room temperature were loaded onto glow‐discharged 200‐mesh carbon‐coated copper grids and incubated for 10 min. The grids were blocked with 0.1% bovine serum albumin (BSA) for 30 min at room temperature, followed by incubation with primary antibodies—Rabbit polyG antibody (PEP122) and Anti‐FLAG M2 antibody—at a dilution of 1:15 for 30 min at room temperature. After washing three times with PBS, the grids were incubated with corresponding 6‐nm colloidal gold‐conjugated secondary antibodies for 30 min at room temperature. Subsequently, the grids were sequentially washed with PBS, double‐distilled H_2_O, and 2% (w/v) uranyl acetate, then stained with 2% (w/v) uranyl acetate for 45 s. Excess liquid was blotted away with filter paper. TEM micrographs were acquired using a Tecnai T12 microscope (FEI) operated at 120 kV.

### 4.7 Cryo‐EM Sample Preparation Data Collection and Image Processing

#### 4.7.1 Sample Preparation

After pronase treatment, 4 µL of the fibril suspension was applied to glow‑discharged holey carbon grids (C‑Flat, 300 mesh, 1.2/1.3 µm; Electron Microscopy Sciences, #71159). Following an 8 s incubation at 16 °C and 100% humidity, excess liquid was blotted off, and grids were plunge‑frozen in liquid ethane using a Vitrobot Mark IV (Thermo Fisher Scientific).

#### 4.7.2 Data Collection

Cryo‑EM data were collected on a 300 kV Titan Krios G4 cryo‑TEM (Thermo Fisher Scientific) equipped with a BioContinuum K3 direct electron detector (Gatan) operating in counting mode and a GIF Quantum energy filter (Gatan; 20 eV slit width) for the removal of inelastically scattered electrons. Super‑resolution movies were recorded at 105 000× magnification (0.83 Å pixel^−^^1^) with a total exposure dose of ∼55 e^−^ Å^−^^2^ over 2.0 s. Automated acquisition was performed using EPU software (Thermo Fisher Scientific) over a defocus range of −1.0 to −2.4 µm.

#### 4.7.3 Image Processing

The 40 movie frames per micrograph were aligned, dose‑weighted, and corrected for beam‑induced motion using MotionCorr2 [77], and subsequently binned to a final pixel size of 0.83 Å. Contrast transfer function (CTF) parameters were estimated from the summed micrographs using CTFFIND‑4.1.8 [78]. Fibrils were manually selected and subjected to reference‑free 2D classification in RELION [79].

### 4.8 Preparation of Acute Brain Slices

Mice received an intraperitoneal injection of THS (5 mg/kg) dissolved in phosphate‐buffered saline containing 10% (w/v) DMSO. Two hours after injection, mice were anesthetized with isoflurane and transcardially perfused with N‐methyl‐D‐glucamine (NMDG)‐HEPES solution containing: 93 mM NMDG, 2.5 mM KCl, 1.2 mM NaHCO_3_, 20 mM HEPES, 25 mM glucose, 5 mM sodium ascorbate, 2 mM thiourea, 3 mM sodium pyruvate, 10 mM MgSO_4_·7H_2_O, and 0.5 mM CaCl_2_·2H_2_O (osmolality 300–315 mOsmol/kg, pH 7.4), as previously described (Preparation of acute brain slices using an optimized N‐methyl‐D‐glucamine protective recovery method). Brains were rapidly removed, and acute slices (120 µm thickness) were prepared using a vibratome (Leica VT1200S; Campden Instruments Limited blades, J52/11SS) at a cutting speed of 0.26 mm/s in ice‐cold carboxygenated NMDG‐HEPES solution.

### 4.9 In‐Grid High Pressure Freezing With Double Sided Attachment Serial Lift‐Out

Tissue preparation for cryo‐electron tomography (cryo‐ET) was adapted from established high‐pressure freezing and lift‐out protocols, with modifications [57]. Acute hippocampal slices from NIID model mice were incubated for 30 min in a cryoprotectant solution (NMDG‐HEPES buffer containing 20% dextran (40,000 MW)). The slices were then mounted onto hydrophilic electron microscopy (EM) grids. These grids were loaded into type B specimen carriers using 2‐methylpentane as a filler, covered with sapphire discs, and immediately high‐pressure frozen using a Leica EM ICE system. Following freezing, the grids were recovered by dissolving the cryoprotectant in ethane at −170°C (EM GP2) and were subsequently stored under liquid nitrogen until further processing.

Cryo‐lamellae were prepared using an Aquilos 2 focused ion beam/scanning electron microscope (FIB/SEM) equipped with an integrated fluorescence light microscope (iFLM). To isolate regions of interest, the workflow began with correlative light and electron microscopy (CLEM) prescreening. A shallow mill (9°) was performed to create a flat reference surface on the tissue grid. The stage was then tilted to 35° for simultaneous cryo‐fluorescence and SEM imaging to precisely locate polyG aggregates.

Once a target was identified, a tissue chunk measuring approximately 40 × 30 µm was isolated through progressive milling (currents decreasing from 15 to 3 nA), creating a freestanding chunk. An adaptor chunk (20 × 15 × 50 µm) was separately milled from the intersection of a receiver‐grid bar, left attached by a thin (∼2 µm) bridge. This adaptor was welded to the EasyLift needle and detached. The adaptor‐equipped needle was then positioned against the isolated tissue chunk, welded, and the chunk was lifted out. For stability during thinning, the lifted chunk was sequentially welded to both sides of the receiver‐grid bars, sectioned into ∼3–4 µm segments, and redistributed. Additional vertical welds were applied to enhance mechanical stability. Finally, lamella milling was performed using automated AutoTEM software, with currents stepwise from 0.5 to 30 pA and a ± 1° tilt compensation. The resulting lamellae were coated with platinum (5 s at 0.20 mbar and 7.0 mA) and were ready for imaging on a 300 keV transmission electron microscope.

### 4.10 Cryo‐ET Data Collection and Processing

For ex vivo aggregates, cryo‐ET data were collected on a Titan Krios cryo‐transmission electron microscope equipped with a Selectris energy filter (20 eV slit width) and a BioContinuum K3 direct electron detector (Gatan). Tilt series were acquired at a calibrated pixel size of 1.37 Å using Tomography (v5.12) in a dose‐symmetric scheme over a tilt range of −60° to 60° with 3° increments. The total accumulated dose was ∼130 e^−^/Å^2^, and the nominal defocus was set to 4 µm. For tissue lamellae prepared from NIID model mice, a correlative cryo‐fluorescence/cryo‐EM workflow was used to localize polyG aggregates on the lamellae prior to tilt‐series acquisition. The initial tilt angle was adjusted according to the lamella pre‐tilt introduced during cryo‐FIB milling in the FIB/SEM; all other acquisition parameters were identical to those used for the ex vivo samples.

For tomographic image processing, raw tilt‐movie frames were motion‐corrected and the CTF was estimated using Warp [80]. Low‐quality tilts were manually excluded. The remaining tilt series were then automatically aligned using patch tracking and reconstructed into tomograms by weighted back‐projection in AreTomo (v2.0) [81]. To improve contrast in regions of interest, CTF deconvolution was performed using IsoNet (v0.2) [82] and IsoNet2 [83].

### 4.11 Tomogram Segmentation and 3D Rendering

For three‐dimensional segmentation of polyG aggregates, a neural network‐based approach implemented in EMAN2 [84] was employed. Briefly, fibrillar or sheet‐like structures identified in both ex vivo and in situ tomograms were manually annotated to generate independent training datasets. These annotations were used to train dedicated neural network models, which were subsequently applied to segment polyG aggregates across multiple tomographic volumes. Cellular membranes were first automatically detected using the MemBrain‐seg package [85] and subsequently refined and analyzed in Amira (Thermo Fisher Scientific). For tomographic reconstructions containing putative proteasome complexes, initial visual inspection was performed to evaluate particle morphology and relative abundance. Template matching of proteasome particles was carried out using PyTom [86], followed by manual false‐positive removal (EMD‐3932 26S proteasome as template). Owing to the limited number of identifiable proteasome particles, systematic coordinate extraction and subsequent sub‐tomogram averaging were infeasible. Thus, coordinate extraction was used only for three‐dimensional visualization of spatial distribution and density map fitting (employing the EMD‐3932 map low‐pass filtered to 30 Å). Final visualization and rendering of segmented aggregates and cellular components were performed using UCSF ChimeraX [87].

### 4.12 In Situ Proximity Labeling Proteomics Analysis

#### 4.12.1 Proximity Labeling

In situ proximity labeling was carried out following a previously established protocol [62]. Frozen brain sections from NIID model mice were fixed, permeabilized, and immunostained to verify the presence of polyG aggregates.

Three consecutive slices from one NIID model mouse were used. As a negative control, sections were incubated without primary antibody (only primary antibody dilution buffer); as the experimental group, sections were incubated with the primary antibody (Flag in primary antibody dilution). All sections were fixed in 10% neutral buffered formalin for 10 min, permeabilized in 0.2% Triton X 100 for 15 min, quenched with 1.5% H_2_O_2_ for 10 min, blocked with goat serum for 1 h, incubated with primary antibody or primary antibody dilution overnight at 4 °C, and then with HRP conjugated secondary antibody for 1 h. For proximity labeling, sections were incubated with reaction buffer (2 µM probe, 10 µM H_2_O_2_) for 5 min at RT, washed with PBST, and then labeled with 40 µM fluorophore azide for 30 min. Then sections were processed according to the click chemistry protocol: CuSO_4_, THPTA, sodium ascorbate solution, and aminoguanidine were sequentially mixed and added to PBS to final concentrations of 1, 5, 15, and 10 mM, respectively.

#### 4.12.2 Protein Extraction and Affinity Enrichment

Labeled tissue sections were lysed in buffer containing 100 µL 1% SDS and 50 mM Tris‑HCl (pH 8.0) and sonicated at 4 °C for 10 min (Diagenode BioRuptor Pico). Lysates were heated at 95 °C for 1 h, sonicated again for 10 min, then mixed with 400 µL SDS‑free RIPA buffer (50 mM Tris‑HCl, pH 8.0, 150 mM NaCl, 0.5% SDC) and clarified by centrifugation (15 000×g, 10 min, 4 °C). For affinity capture, 2 µL Streptavidin Sepharose was washed twice with RIPA (50 mM Tris‐HCl, pH 8.0, 150 mM NaCl, 0.5% SDC, 0.2% SDS) buffer and once with SDS‑free RIPA. Clarified lysate was added to the beads and incubated overnight at 4 °C with gentle rotation. Beads were washed sequentially with: 1% SDS (3 times), 1 M NaCl (1 time), 10% ethanol (3 times), and 50 mM ABC (1 time). Reduction and alkylation were done in 10 mM TCEP, 40 mM chloroacetamide in 50 mM ABC at 37 °C for 30 min, followed by a 50 mM ABC wash. Beads were resuspended in 20 µL digestion buffer (0.02 µg/µL trypsin; Promega, V5111) and incubated overnight at 37 °C. Digestion was stopped with 1% formic acid, and peptides were desalted on C18 StageTips.

Mass spectrometry data acquisition, processing and bioinformatics analysis. The resulting peptides were analyzed by LC‐MS/MS on a Q Exactive HF‐X mass spectrometer coupled to an EASY‐nLC 1200 system, operating in direct data‐independent acquisition (directDIA) mode. Raw data were processed using Spectronaut software (Biognosys) against the UniProt Mus musculus reference proteome (UP000000589, 17 223 entries, downloaded in February 2025) derived from reviewed Mus musculus entries in UniProt. Proteomics data were analyzed using Perseus (Version 2.0.11) and visualized by Rstudio. First, the PG.Quantity (intensity), PG.Organisms and PG.Genes were loaded into Perseus. Rows were filtered based on a categorical column and only entries corresponding to Mus musculus were kept. After adding categorical annotation rows and filtering rows based on valid values (requiring at least 3 valid values in one group), normalization was performed using endogenous biotinylation correction for experimental vs. control comparisons. The datasets were then log2‑transformed, and missing values were imputed using a width of 0.3 and a downshift of 1.8. For the analysis of the enriched proteome, Student's t‑test was applied with thresholds of p‑value < 0.05 and log2(fold change) > 1, followed by the generation of volcano plots.

Proteins associated with the “proteasome complex” are highlighted in magenta, those associated with “protein folding and chaperonin” are shown in green, and previously reported polyG‐interacting proteins are highlighted in purple. Functional enrichment analysis of Gene Ontology Biological Process (GOBP) terms was conducted using the clusterProfiler package, employing a one‐tailed Fisher's exact test followed by Benjamini–Hochberg correction for multiple testing. Additionally, KEGG pathway enrichment analysis was performed to identify significantly enriched signaling and metabolic pathways.

## Author Contributions

C.L. and Q.L. conceived the project and supervised all research. H.D., Y.P., Z.T, and Y.Y. conducted the main experiments and data analysis. Z.T. and H.X. assisted with mass spectrometry sample preparation and data analysis, and G.Z. and H.D. performed Cryo‐ET experiments and data processing. J.W., Y.T., and B.T. provided patient skin biopsy samples. C.L., Q.L., R.T., Q.G., and D.L. interpreted the data and revised the manuscript. H.D. and C.L. wrote the manuscript with inputs from all authors.

## Conflicts of Interest

The authors declare no conflict of interest.

## Supporting information




**Supporting File 1**: advs76630‐sup‐0001‐TableS1.xlsx.

**Supporting File 2**: advs76630‐sup‐0002‐MovieS1.mp4.

**Supporting File 3**: advs76630‐sup‐0003‐MovieS2.mp4.

**Supporting File 4**: advs76630‐sup‐0004‐SuppMat.docx.

## Data Availability

The data that support the findings of this study are available from the corresponding authors upon reasonable request.
