# Supplementary material for: PolyG Fibrils Coalesce Into Nuclear Ribbons That Engage Proteostasis Machinery in Neuronal Intranuclear Inclusion Disease
Source: Adv Sci (Weinh). 2026 Jul 20;13(56):e76630. doi: 10.1002/advs.76630 (PMC13383697; doi:10.1002/advs.76630)
Supplement: Supplementary file 1 [file ADVS-13-e76630-s002.docx]

**Supplementary Figures**

**Figure S1. Purification and characterization of polyG aggregates from NIID mouse brain.**

(A) Schematic protocol for the purification of polyG aggregates from NIID mouse brain.

(B) Western blot analysis of different fractions collected during the purification process, probed with anti‑FLAG antibody.

(C) Representative negative‑stain transmission electron microscopy (TEM) images of the precipitated fraction, showing polyG aggregates. Scale bar: 200 nm.

(D) Representative negative‑stain TEM images of polyG aggregates labeled with immunogold nanoparticles (anti‑FLAG and anti-PEP122). Scale bar: 20 nm.

**Figure S2. Cryo**‑**EM characterization of polyG aggregates purified from NIID mouse brain.**

(A) Representative cryo‑EM images of purified polyG aggregates, illustrating the heterogeneous morphology. Red arrows: loosely organized coarse fibrils; blue arrows: clustered fibrils; yellow arrows: clustered fine fibrils. Scale bar: 100 nm.

(B) Representative 2D class averages of polyG fibrils and their corresponding fast Fourier transforms (FFTs), revealing a weak but detectable signal at approximately 4.7 Å. Scale bar: 10 nm.

(C) Representative cryo‑EM images and corresponding power spectra of a sample containing polyG fibrils (left) and a collagen‑only control (right). The power spectrum of the polyG‑containing sample shows a distinct 4.7 Å band (arrow), which is absent in the control spectrum. Scale bar: 50 nm in Cryo-EM micrographs.

**Figure S3. Characterization and quantification of branched fibrils in brain-extracted polyG aggregates.**

(A) Representative Cryo-ET slice showing branched fibrils in the purified aggregate population. Branch points are highlighted by red arrows. Scale bar: 50 nm.

(B) Quantification of the branch width distribution.

See also Movie S1.

**Figure S4. Cryo**‑**ET characterization of ex-vivo polyG aggregates.**

(A) Representative cryo‑ET slice showing the morphological diversity of purified aggregate populations. Scale bar: 100 nm.

(B) Quantification of the proportions of distinct aggregate morphologies observed in (A), based on tomogram statistics.

**Figure S5. Characterization of polyG inclusions in NIID mouse brain using molecular probe staining.**

(A) Immunofluorescence staining of polyG inclusions in NIID mouse brain using classical or previously reported amyloid probes, including ThS, Congo Red, APN1607 (reported tau tracer), BF227 (reported Aβ tracer), and PBB3 (reported tau tracer). Scale bar: 25 µm (overview), 5 µm (inset).

(B) Quantification of colocalization between each small‑molecule probe and polyG inclusions shown in (A). Pearson’s correlation coefficients indicate that all tested probes label polyG inclusions in NIID brain to varying degrees.

(C) Comparative staining of ThS in 5×FAD mouse brain (Aβ plaques) and NIID mouse brain (polyG inclusions). Representative images are shown. Scale bar: 100 µm (overview), 20 µm (inset).

(D) Quantification of ThS‑positive signals (based on colocalization area) in Aβ plaques versus polyG inclusions, confirming differential labeling efficiency.

**Figure S6. *In situ* cryo-ET characterization of polyG aggregates.**

(A) Representative low‑magnification cryo‑correlative light and electron microscopy (cryo‑CLEM) image of a polyG inclusion within NIID mouse brain tissue. Scale bar: 500 nm.

(B) Tomographic slice (left) and corresponding 3D segmentation (right) showing the *in tissue* architecture of polyG ribbons. PolyG aggregates are colored cyan. Scale bar: 25 nm.

**Figure S7. Cryo**‑**ET reconstruction reveals interactions between polyG ribbons and membrane vesicles, related to Figure 4.**

(A) Representative tomographic slice showing polyG ribbons and surrounding vesicles. Scale bar: 100 nm.

(B) Multi‑Z series of the boxed region in (A); Z‑layer information is indicated in the figure. Scale bar: 20 nm.

(C–G) Representative views from different tomograms showing direct contacts between polyG ribbons and cellular membranes. Scale bar: 20 nm (for each panel).

(H) A rare view capturing dense polyG aggregates within a multilamellar vesicle. Scale bar: 20 nm.

**Figure S8. Validation of proximity labeling specificity for polyG aggregate-associated proteomics.**

(A) Low-magnification immunofluorescence images of NIID mouse brain sections. Scale bar: 500 μm.

(B) Higher-magnification views of the boxed regions in (A), showing specific biotinylation signals (streptavidin, red) surrounding polyG aggregates (anti-Flag, green) only under antibody-added conditions. Right panel: Quantification of colocalization between polyG aggregates and streptavidin signals, expressed as Pearson's correlation coefficient. Scale bar: 25 μm.

**Figure S9. Colocalization of proteasome components with polyG aggregates in NIID patient skin and mouse brain tissues.**

(A) Immunofluorescence micrograph of a skin biopsy from a NIID patient, showing colocalization of the proteasome subunit PSMC6 (red) with polyG aggregates (green). Nuclei are counterstained with DAPI (blue). Scale bar: 10 μm.

(B) Immunofluorescence micrograph of an NIID mouse brain section, demonstrating colocalization of PSMC6 (red) with intranuclear polyG inclusions (anti-Flag, green). Nuclei are labeled with DAPI (blue). Scale bar: 5 μm.

(C) Higher-magnification showing polyG aggregates at distinct subcellular localizations. Colocalization with PSMC6 is observed in both extranuclear (top) and intranuclear (bottom) regions. Scale bar: 2 μm.

**Figure S10. Volcano plot of proximity proteomics data cross**‑**validating previously reported polyG**‑**interacting proteins.** Previously reported polyG‑interacting proteins are highlighted in purple on the volcano plot (left panel). Significance thresholds: p‑value < 0.05 and |log₂(fold change)| > 1. The right panel lists the validated interactors and their corresponding statistical metrics.

**Figure S11. Characterization of proteasome**‑**like particles within polyG inclusions.**

(A) Representative tomographic slice showing 10 nm proteasome‑like particles enriched around polyG ribbons. Colored boxes indicate regions shown as multi‑Z slices, including a single‑capped proteasome (orange), a 10 nm ring‑like particle (top view of a proteasome, blue), and a double‑capped 26S proteasome (red and brown). Scale bar: 50 nm.

(B) Representative tomographic slice showing the fitting of an un‑averaged density map of a proteasome‑like particle (directly extracted from the denoised tomogram) to a proteasome density map (inset). Scale bar: 50 nm (tomogram); 5 nm (enlarged view).

**Movie S1. Cryo-ET of purified polyG aggregates, Related to Figure S3.**

**Movie S2. Cryo-ET of in tissue polyG inclusions, Related to Figure 5.**

**Table S1. Proximity labeling–based proteomic analysis of polyG aggregates in the NIID mouse brain.**
